# Ontario Veterinary College, Toronto, Canada

**Published:** 1894-12

**Authors:** 


### ONTARIO VETERINARY COLLEGE, TORONTO, CANADA.

The Christmas examinations of this well known and prosperous institution were concluded on Friday, December 21st. The Board of Examiners, which is appointed by the Board of Agriculture and Arts of the Province of Ontario, subjected the aspirants for the diplomas which they grant to a searching examination.

The following gentlemen were successful in passing the ordeal :

| | |
|---|---|
| Ball, Nelson, - - - | Auburn. |
| Coleman, David H., - - - | Philadelphia, Pa. |
| Cunningham, Robert, - - | Thorndale. |
| Dunn, George H., - - - | Erie. |
| Elliot, John T., - - - | Uxbridge, |
| Engal, John H., - - - | Milverton. |
| Friedheim, Louis, - - - | Rock Hill, S. C. |
| Funston, William H., - - - | Toronto. |
| Harrison, G. W., - - - | Souris, Man. |
| Hisey, Dan, - - - - | Creemore. |
| Irvine, John D., - - - | Dalkeith. |
| Jemison, Geo., - - - - | North Bay. |
| Kiethline, John T., - - | Jenningsville, Pa. |
| McAfee, Robert A., - - - | Aylwin, Que. |
| Macdonald, John H., - - | Wiarton. |
| Sawyer, S. Gordon, - - - | Boston, Mass. |
| Snider, J. Herbert, - - | Guelph. |
| Swenerton, Wilson, - - - | Wawanesa, Man. |
| Thomlinson, Benjamin, - - | Clinton. |
| Whybra, Farnell, - - - | Niagara Falls, N. Y. |

Primary examination—Arthur P. Freeman, anatomy and materia medica.

